# Exopolysaccharide Biosynthesis Enables Mature Biofilm Formation on Abiotic Surfaces by *Herbaspirillum seropedicae*

**DOI:** 10.1371/journal.pone.0110392

**Published:** 2014-10-13

**Authors:** Eduardo Balsanelli, Válter Antonio de Baura, Fábio de Oliveira Pedrosa, Emanuel Maltempi de Souza, Rose Adele Monteiro

**Affiliations:** Department of Biochemistry and Molecular Biology, Universidade Federal do Paraná, Curitiba, Paraná, Brazil; National Institutes of Health, United States of America

## Abstract

*H. seropedicae* associates endophytically and epiphytically with important poaceous crops and is capable of promoting their growth. The molecular mechanisms involved in plant colonization by this microrganism are not fully understood. Exopolysaccharides (EPS) are usually necessary for bacterial attachment to solid surfaces, to other bacteria, and to form biofilms. The role of *H. seropedicae* SmR1 exopolysaccharide in biofilm formation on both inert and plant substrates was assessed by characterization of a mutant in the *espB* gene which codes for a glucosyltransferase. The mutant strain was severely affected in EPS production and biofilm formation on glass wool. In contrast, the plant colonization capacity of the mutant strain was not altered when compared to the parental strain. The requirement of EPS for biofilm formation on inert surface was reinforced by the induction of *eps* genes in biofilms grown on glass and polypropylene. On the other hand, a strong repression of *eps* genes was observed in *H. seropedicae* cells adhered to maize roots. Our data suggest that *H. seropedicae* EPS is a structural component of mature biofilms, but this development stage of biofilm is not achieved during plant colonization.

## Introduction

*H. seropedicae* is a nitrogen-fixing, plant-growth-promoting Betaproteobacterium found attached to and within tissues of important crops such as maize (*Zea mays*), rice (*Oryza sativa*), sorghum (*Sorghum bicolor*) sugar-cane (*Saccharum officinarum*) and wheat (*Triticum aestivum*) [1]. The molecular mechanisms of plant recognition, attachment, penetration and endophytic colonization of this microrganism are not well known [1]. EPS are carbohydrate polymers of highly variable composition and structure found outside cells [2]. Bacterial EPS are usually responsible for attachment to solid surfaces and to other bacteria, thus forming microscopic and macroscopic cell aggregates [3]. When the aggregates are neatly organized, they are called biofilms [4]. In these communities the surface-associated microorganisms grow in matrix-enclosed microcolonies separated by a network of open-water channels [5], [6]. The presence of a matrix between cells confers a series of selective advantages, such as protection against environmental variations, nutrient and ions retention, resistance to desiccation and mechanical protection [4], [7], [8].

Most of microorganisms do not occur naturally in planktonic communities, being generally found attached to biological and non-biological surfaces forming biofilms [9]. Initial stages of biofilm formation involves the redistribution of attached cells by surface motility [10]–[12], binary division of attached cells [13] or recruitment of cells from the surrounding fluid to the developing biofilm [14]. The individual adherent cells that initiate biofilm formation on a surface are capable of independent movement [12] before they begin to exude exopolysaccharide and adhere irreversibly [5]. Biofilm maturation results in the generation of a complex architecture with channels, pores, and redistribution of bacteria away from the substrate [15]. As the biofilm matures many cells alter their physiological processes in response to the conditions in their particular niches. The biofilm cells express genes in a pattern that deeply differs from that of their planktonic counterparts [16]. Finally, individual cells or whole microcolonies may detach from the biofilm and colonize other surfaces [17].

EPS and biofilm formation have been associated with the capacity of bacteria to colonize plants in symbiotic, neutral or pathogenic associations. One of the EPS functions in plant-bacterial interaction is to permit epiphytic colonization of the plant host [18]. Also, in plant-pathogen interaction EPS helps to create a favorable environment for pathogen survival and growth inside the infected plant, acting as a protective barrier against plant metabolic defenses [19]. The knockout of EPS biosynthesis genes (*exo* or *eps*) resulted in loss of virulence by *Erwinia stewartii* and *Xanthomonas axopodis* [20]. The mutation of *Xanthomonas campestris gumD*, which codes for a glucosyltransferase, drastically decreased the pathogenicity of this organism [21]. Also, EPS was the main factor required for bacterial wilt caused by *Ralstonia solanacearum* [22], where it seems to interfere with plant water transport by clogging the xylem [23]. In the case of diazotrophic symbionts, EPS seems to be indispensable for functional nodule establishment [24], [25]. *Ensifer meliloti* mutant strains deficient in the production of one kind of EPS induce nodule formation, but they do not contains bacteroids [26], [27]. The knockout of acidic EPS biosynthesis genes of *Ensifer* sp. NGR234 also results in pseudonodule formation [28]. The infection and subsequent nodulation of legumes by *R. leguminosarum* requires bacterial attachment onto root hair, a process that involves EPS production [29]. In the case of associative diazotrophs such as *Azospirillum brasilense* and *Gluconacetobacter diazotrophicus*, EPS seems to influence cellular aggregation and biofilm formation on plant root surface [30]–[32]. The knockout of rhamnose biosynthesis in *A.* *brasilense* led to a decrease in EPS production, and a decrease in maize colonization [33]. In *G. diazotrophicus,* exopolysaccharides seem to have a more dramatic effect, where knockout of *gumD* abolished attachment to rice root surface and endophytic colonization [32].

There is no evidence of the role of *H.* *seropedicae* EPS in plant colonization, although scanning electron microscopy revealed production of mucilaginous and fibrillar materials by *H. seropedicae* during colonization of maize, rice and sorghum root surfaces [34], [35]. This material might be EPS. In this work we knocked out the *epsB* gene which codes for a putative glucosyltransferase of the EPS biosynthesis gene cluster of *H. seropedicae*. The mutant strain has diminished EPS production and biofilm formation on abiotic surfaces, but showed no alterations on maize colonization profile compared to the wild type.

## Materials and Methods

### Growth of bacterial strains, DNA manipulations and mutagenesis

Bacterial strains and their relevant characteristics are listed in Table 1. *Herbaspirillum seropedicae* strains were grown at 30°C and 120 rpm in NFbHPN medium [41]. *Escherichia coli* strains were grown at 37°C in LB medium [42]. Antibiotics were added at the following concentrations when required: ampicillin (Ap) 10 µg.mL^−1^; kanamycin (Km) 50 µg.mL^−1^; chloramphenicol (Cm) 30 µg.mL^−1^; tetracycline (Tc) 10 µg.mL^−1^; streptomycin (Sm) 80 µg.mL^−1^. The plasmids used in this study are listed in Table 1. Plasmid and total DNA preparations, agarose gel electrophoresis, restriction endonuclease digestion and cloning were performed according to standard protocols [42].

**Table 1:** Bacterial strains and plasmids used in this study.

Strains	Relevant characteristics a	Reference
*E. coli* Top 10	F^−^ *mcrA Δ(mcrr-hsd*RMS*-mcr*BC*) φ*80*lacZΔ*M15 *ΔlacX*74 *ara Δ*139 *Δ(ara,leu)* 7697* nup*G *λ* ^−^	Invitrogen
*E. coli* S17.1	RP4-2-*Tc::Mu-Km::Tn7*	[36]
*H. seropedicae* SmR1	Spontaneous Sm^r^ derived from strain Z78 (ATCC 35893)	[37]
*H. seropedicae* EPSEB	*epsB* mutant, Sm^r^, Km^r^	This work
*H. seropedicae* MHS01	*epsG::lacZ* chromosomal reporter fusion, Sm^r^, Km^r^	[38]
*H. seropedicae* SmR1+pHC60	*H. seropedicae* SmR1 constitutively expressing GFP from pHC60, Sm^r^, Tc^r^	This work
*H. seropedicae* EPSEB+pHC60	*H. seropedicae* EPSEB constitutively expressing GFP from pHC60, Sm^r^, Km^r^, Tc^r^	This work
**Plasmids and vectors**		
pTZHSepsB	pTZ57 containing *H. seropedicae* SmR1 *epsB* gene, Ap^r^	This work
pTZHSepsBKM	pTZHSepsB with *epsB* gene disrupted by Tn5 Kan cassette, Ap^r^, Km^r^	This work
pSUPHSepsBKM	*epsB* gene disrupted by Tn5 Kan cassette inside Tc gene of pSUP202, Ap^r^; Km^r^; Cm^r^;	This work
pUC4-KIXX	Ap^r^; Km^r^; cassette Tn5 Kan	[39]
pSUP202	Ap^r^; Tc^r^; Cm^r^; mob site	[36]
pTZ57R/T	Ap^r^, TA cloning vector	Fermentas
pHC60	Tc^r^; constitutive GFP (GFP-S65T) expression	[40]

a Ap = ampicillin; Km = kanamycin; Sm = streptomycin; Tc = tetracycline; Cm = chloramphenicol; and the superscript r = resistant.

For *epsB* mutagenesis the primers HSepsB-F (5′- gctggaaccgcatatgatcgt-3′) and HSepsB-R (5′- ccaggtggatccggtcaataa-3′) were used to amplify the *epsB* gene from *H. seropedicae* genomic DNA, and the amplicon was cloned in pTZ57R/T. The generated plasmid pTZHSwaaL was disrupted in the EcoRV site by the *nptI* cassette isolated from pKIXX that confers resistance to kanamycin (Km). The disrupted gene was transferred to pSUP202. This construction was electro-transformed in *E. coli* S17.1, and the transformants were conjugated into *H. seropedicae* SmR1. The mutant strains were selected and named *H. seropedicae* EPSEB (*epsB* ^−^ *).* Insertion of the cassette in the genome of the mutant strain by double crossover event was confirmed by PCR analyses. Wild-type and EPSEB mutant strains were GFP-marked through conjugation with *E. coli* S17.1 harboring the pHC60 plasmid.

### EPS and LPS analyses

For EPS extraction, the *H. seropedicae* wild type and EPSEB mutant strains were grown in 10 mL of NFbHPN medium [41] at 30C and 120 rpm in the presence of 50 mg of sterile glass fiber. After 12hours, the bacterial cultures together with the glass fiber were transferred to a 50 mL centrifuge tube, and vortexed vigorously for 1 minute to remove glass fiber attached bacteria. The cells and the glass fiber were then removed by centrifugation (15 min, 3000 g) and the supernatant was filtered through a 0.22 µm membrane to remove residual cells. Exopolysaccharides in the filtered supernatant were precipitated with 3 volumes of cold ethanol for 24hours at 20C and centrifuged for 10 minutes at 4°C and 3000 g. The precipitate was vacuum dried, resuspended in MilliQ water and dialyzed against MilliQ water. Ten microliters of dialyzed samples were mixed with sample buffer (120 mM Tris pH 6.8; 3% SDS; 9% β-mercaptoethanol; 30% glycerol; 0.03% bromophenol blue), separated by SDS-PAGE (12% acrylamide) and visualized by silver periodate oxidation staining [43]. Total sugar concentration of the samples was determined with phenol/sulfuric acid [44], using glucose as standard.

LPS extraction for electrophoretic analysis was performed according to Balsanelli *et al.* [45] by the proteinase K – SDS method. Four microliters of final mixture were separated by SDS-PAGE (16% acrylamide) and visualized by silver periodate oxidation staining [43].

### Biofilm formation on glass fiber

*H. seropedicae* strains were grown as described for EPS isolation, and biofilm formation was evaluated according to Balsanelli et al. [45]. Briefly, twelve hours after inoculation glass fiber was removed from the medium, stained with 20 µL of crystal violet 1% for 2 minutes, and washed three times with 0.9% saline solution. Then, 1 mL of absolute ethanol was added to remove the dye, and the alcoholic solution was used to determine the OD_550_. The values are expressed as OD_550_ of the samples subtracted from the OD_550_ of the fiber glass treated culture medium. The results reported represent the average of three independent experiments. Purified wild type EPS (100 µg of glucose equivalents.mL^−1^) was added to the system during incubation with glass fiber to test complementation of the mutant strain phenotype. Samples of stained glass fibers were analyzed by light microscopy for visualization of biofilm structure.

### Plant interaction assays

Assays of maize colonization by *H. seropedicae* strains were performed according to Balsanelli *et al.* [46]. Briefly, seeds of *Zea mays* cv. 30F53, *Oryza sativa* cv Nipponbare or *Sorghum bicolor* cv A07 were surface-sterilized, germinated and each seedling was inoculated with 10^5^ CFU of *H. seropedicae* strains. The inoculated seedlings were transferred to a hydroponic system containing 30 mL of plant medium [47] and 10 g of sterile culture beads in 100 mL glass tubes. Bacterial counts were made immediately after inoculation to access attached bacteria and 1, 4, 7 or 10 days after inoculation to access endophytic and epiphytic bacteria. The results reported represent the average of at least three independent experiments.

The GFP-marked strains were used as inoculants as described above, and longitudinal root cuts were freshly prepared for visualization. Root attached and 7 d.a.i. epiphytic bacteria were visualized by confocal laser scanning microscopy (CLSM) on a Nikon Ti Microscope. Plant tissues showed DAPI autofluorescence. Snapshots of the tridimensional images were obtained with the NIS-Elements software (Nikon).

Competition assays were performed using as inoculant a mixture of *H. seropedicae* wild type and *epsB* strains in 1∶1 proportion, with a total of approximately 10, 10^2^, 10^3^, 10^4^, or 10^5^ bacteria per seedling. Total bacterial counts were made as described before, and the strains were identified by antibiotic resistance.

### Chemical resistance assays

Resistance to chemical compounds by *H. seropedicae* strains was determined by serial dilution of liquid cultures and microdrop plating on solid NFbHPN medium containing naringenin (0–250 µM), quercetin (0–250 µM), jasmonic acid (0–10 µM), salicylic acid (0–50 µg.mL^−1^), sodium dodecyl sulphate (0–0.01% w/v) or phenol (0–1% w/v). Data were expressed as percentage of colony forming units in the test plates compared to the control after 24 hours of growth at 30°C.

### EPS biosynthesis gene expression during rhizoplane colonization and biofilm formation

To evaluate *eps* gene expression during rhizosphere colonization, the *H. seropedicae* MHS01 [38] (*epsG::lacZ*) reporter strain was grown in NFbHPN medium for 16 h. After adjusting the culture to OD_600_ = 1.0 in saline buffer, 10^8^ cells (1 mL) were inoculated onto maize in the hydroponic system described above and incubated at 28°C. After 24 h, bacterial cells were recovered from the liquid medium by centrifugation and attached cells were recovered from root surface and polypropylene spheres by vortexing and centrifugation.

To evaluate *eps* gene expression during biofilm formation, the *H. seropedicae* MHS01 reporter strain was grown in the presence of glass fiber as described. After 12 h of growth the free living cells were recovered by centrifugation and attached cells were recovered from glass fiber by vortexing and centrifugation. The β-galactosidase activity of the recovered cells was then measured [48]. Protein determination was carried out according to Bradford [49]. The β-galactosidase activity is reported as nmol of o-nitrophenol produced per minute and mg of protein. The results reported represent the average of at least three independent experiments. The control containing uninoculated maize seedlings did not show any detectable β-galactosidase activity.

## Results

### Genomic organization of *H. seropedicae* EPS biosynthesis genes and mutagenesis

Analyses of *H. seropedicae* SmR1 genome sequence (CP002039) showed a cluster of 28 genes that code for proteins probably involved in the biosynthesis and secretion of EPS (Fig. S1). The organization of these genes is highly similar to the *eps* cluster of *Herminiimonas arsenicoxydans* [50] and *Methylobacillus* sp. 12S [51], and the encoded proteins share high identity to the homologous proteins of all three microorganisms (Table S1). The EPS produced by *Methylobacillus* sp. 12S, named metanolan, is a heteropolymer composed of glucose, galactose and mannose in a 3∶1:1 molar proportion [52]. The analyses of *H. seropedicae eps* genes that code for glycosyltransferases and sugar modifying proteins (such as epimerases and phosphatases in Table S1) suggest that the EPS is composed of these same monosaccharides. Indeed, monosaccharide composition analysis of *H. seropedicae* Z67^T^ EPS showed galactose, glucose and mannose as constituents at a proportion of 4∶3:1, with possible substitutions with tetracarboxylic acids [53].

### Knockout of *epsB* strongly reduces EPS production by *H. seropedicae*

**Figure 1:**
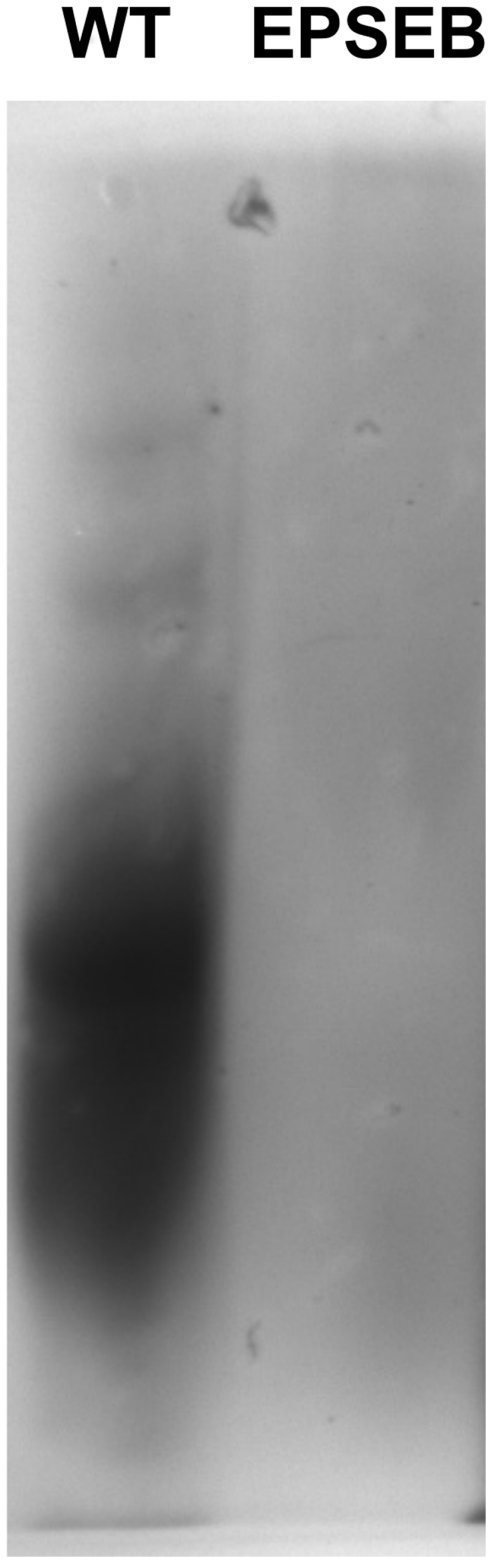
Electrophoretic pattern of EPS isolated from *H. seropedicae* strains SmR1 (wild type) and EPSEB (*epsB* mutant). SDS-PAGE was performed with EPS extracted by cold ethanol precipitation of the supernatant of biofilm growing bacteria in glass fiber submersed in NFbHPN medium.

The production of EPS was initially evaluated by precipitation of *H. seropedicae* strain culture supernatant with 3 volumes of cold ethanol. When the wild type and EPSEB (*epsB*) strains were grown in liquid NFbHPN for 24h no EPS was produced in the culture supernatant. Since in many bacteria EPS biosynthesis is induced during biofilm formation [54], the supernatant of *H. seropedicae* wild type culture grown for 12hours in the presence of glass fiber was processed as above and 0.8 mg.mL^−1^ of EPS was obtained. In contrast with the wild type strain, no EPS could be detected from the EPSEB strain. The samples were then analyzed by a 12% SDS-PAGE (Fig. 1). Exopolysaccharide from the wild type strain showed three poorly defined bands of different molecular weight/charge, while supernatant of EPSEB strain had no polysaccharide band.

The EPSEB strain LPS electrophoretic profile did not differ from that of the wild type (Fig. S2), suggesting that this glucosyltransferase is specific for EPS biosynthesis.

### *H. seropedicae* EPS is necessary for biofilm formation on glass fiber

**Figure 2:**
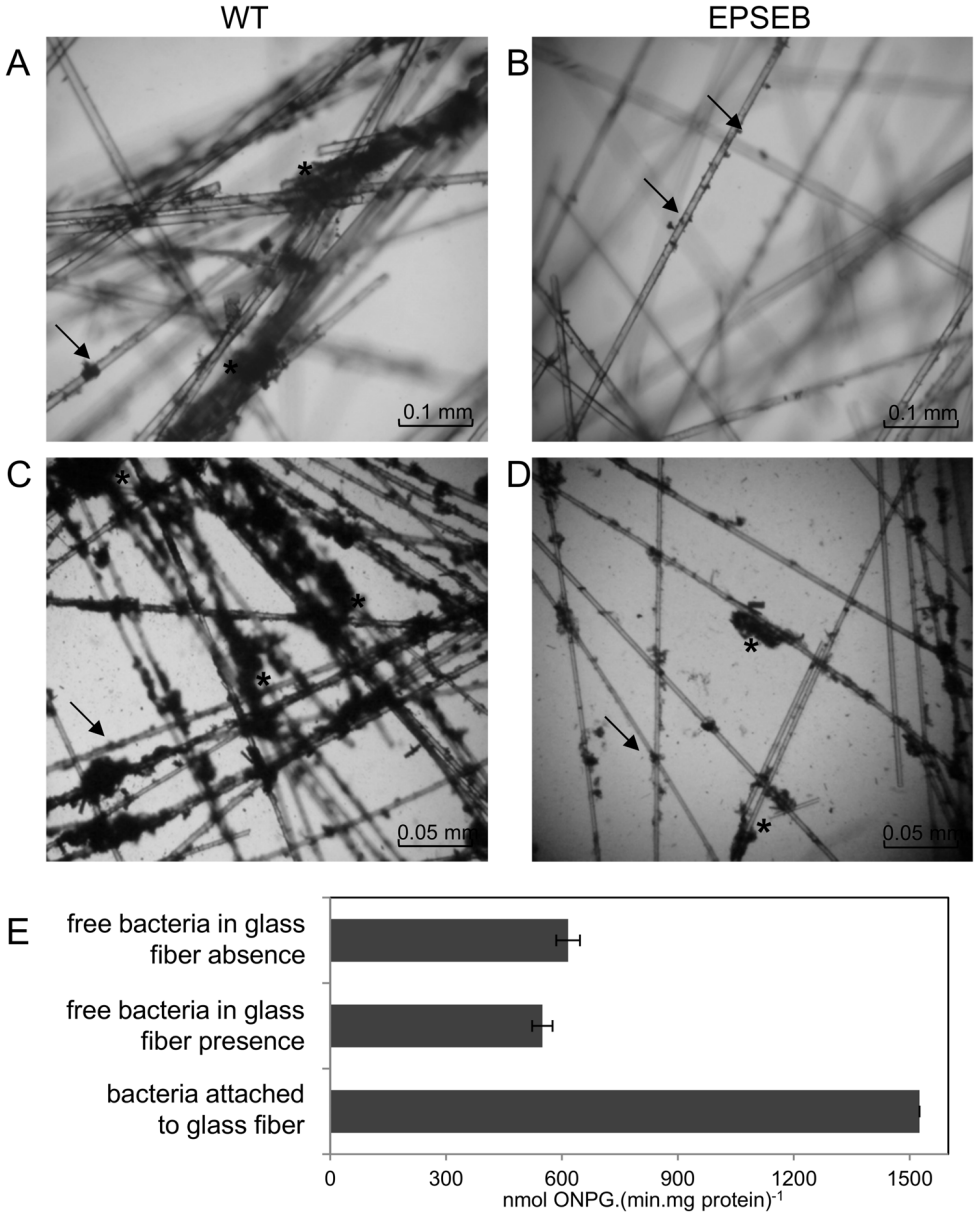
*H. seropedicae* biofilm formation on glass fiber. Light microscopy was performed with *H. seropedicae* SmR1 and EPSEB (*epsB* mutant) grown in the presence of glass fiber for 12 hours, without (A,B) and with (C,D) addition of purified wild-type EPS (100 µg.mL^−1^). Arrows indicate attached bacteria. Asterisks indicate mature biofilm colonies. For biofilm expression analyses (E), *H. seropedicae* MHS-01 cells were grown for 12 h in the presence or absence of glass fiber, the free living bacteria were directly used and biofilm bacteria were recovered from glass fiber by vortex. β-galactosidase activity was determined, standardized by total protein concentration, and expressed as nmol ONP.(min.mg protein) ^−1^± standard deviation. Different letters indicate significant differences (p<0.01, Duncan multiple range test) in *epsG* expression between the tested conditions.

To evaluate the role of EPS in biofilm formation, the strains were grown in the presence of glass fiber and biofilm formation was evaluated quantitatively by staining attached bacteria (Table 2), and qualitatively by light microscopy (Fig. 2A–D). After twelve hours of growth the EPSEB strain showed a 45% reduction in biofilm formation compared to the wild type. Furthermore, microscopic observation showed that the wild type strain formed large tridimensional structures, considered as mature biofilms (Fig. 2A). On the other hand, the mutant strain did not form mature biofilms, with only few attached cells (Fig. 2B). This phenotype was partially restored by the addition of purified *H. seropedicae* EPS (Fig. 2D), suggesting that this polysaccharide is required for biofilm development.

**Table 2:** *H. seropedicae* EPS is required for biofilm formation on glass fiber.

Strains	Biofilm in glass fiber (O.D.550_nm_)	Biofilm in glass fiber+wild-type EPS (O.D.550_nm_)
*H. seropedicae* SmR1	0.66±0.02 a	0.67±0.02 a
*H. seropedicae* EPSEB	0.30±0.01 b	0.54±0.03 c

*H. seropedicae* strains were grown in the presence of glass fiber and purified wild type EPS (100 µg.mL^−1^) when indicated. After 12 hours, bacteria attached to the fiber were stained with crystal violet, washed and de-stained with absolute ethanol. The absorbance of the ethanol (550 nm) was determined and subtracted from the absorbance of the control without bacteria. Different letters indicate significant difference (p<0.001, Duncan multiple range test) between biofilm formation by the strains.

The reporter strain MHS01 (*epsG::lacZ*) was used to determine the regulation of *eps* genes in glass fiber biofilm formation (Fig. 2E). After 12hours of growth in the above-described system, *epsG* expression in glass fiber attached cells was about 3 times higher than in planktonic cells. The *eps* genes up-regulation on bacteria adhered to glass fiber suggests the involvement of EPS in biofilm formation on inert matrix.

### Maize colonization by *H. seropedicae* is not dependent on EPS production

**Figure 3:**
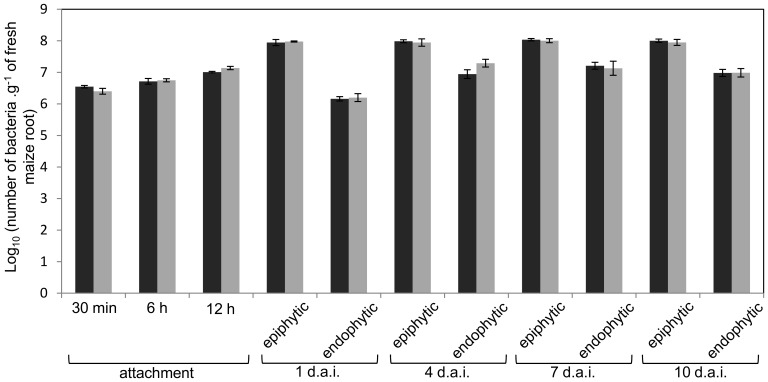
Maize root colonization by *H. seropedicae* wild type (black bars) and *epsB* (gray bars) mutant strain. Results are shown as average of Log_10_ (number of bacteria.g^−1^ of fresh root) ± standard deviation. d.a.i. = days after inoculation.

**Figure 4:**
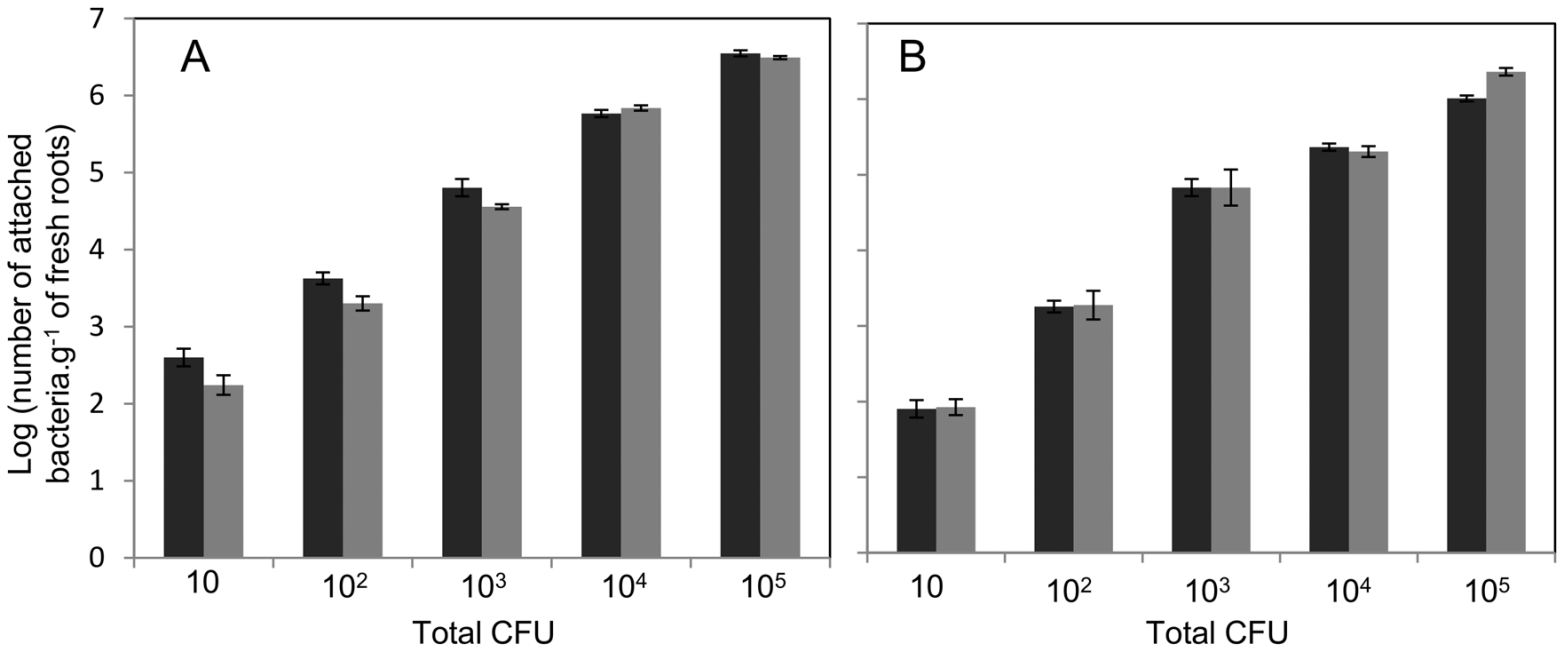
*H. seropedicae* strains competition for attachment on maize roots. *H. seropedicae* wild type (black bars) and *epsB* ^−^ (gray bars) strains were inoculated on maize separately (A) or co-inoculated in a 1∶1 proportion (B), with the total of bacteria inoculated per plantlet indicated in the x axis. Results are shown as average of Log_10_ (number of recovered attached bacteria.g^−1^ of fresh root) ± standard deviation, CFU = colony forming units.

Colonization of *H. seropedicae* strains on maize roots was followed to evaluate the role of EPS in this interaction. The colonization profile of the EPSEB strain was very similar to that of the wild type (Fig. 3), suggesting that attachment, epiphytic and endophytic colonization are not dependent on *epsB* gene. Colonization of rice and sorghum by the EPSEB strain was also very similar to that of the wild type strain (Fig. S3), suggesting that EPS production is not required for interaction with poaceous plants. The maize colonization profile of MHS01 was also similar to the wild type one [48], indicating that the *eps* gene cluster and its product are not involved in plant interaction. The use of smaller numbers of wild type and EPSEB cells in attachment assays on maize roots did not show differences of colonization between the strains (Fig. 4).

**Figure 5:**
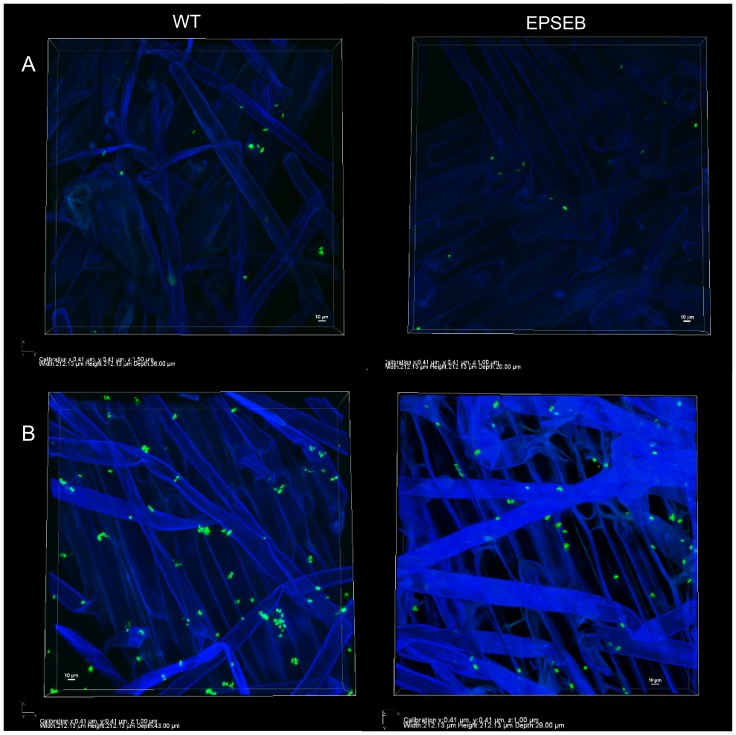
*H. seropedicae* attachment and epiphytic colonization of maize roots. *H. seropedicae* SmR1+pHC60 (GFP- wild type) and EPSEB+pHC60 (GFP- *epsB* mutant) strains were inoculated on maize, and immediately after inoculation (A) or 7 days after inoculation (B), longitudinal samples of the roots were analyzed by laser scan confocal microscopy. Legends under the figures show positioning coordinates of the tridimensional images.

CLSM analyses showed that both wild type and *epsB* mutant strains attach onto the maize root epidermis and root hair as individual cells and in similar numbers (Fig. 5A). Seven days after inoculation (Fig. 5B), the epiphytic population of both strains was still formed of individualized cells, not comprising tridimensional biofilm structures. These results indicate that *H. seropedicae* do not develop mature biofilms on roots as observed on glass fiber, stressing that EPS production is not required in plant colonization.

### *H. seropedicae* EPS is required for resistance to abiotic stress

**Figure 6:**
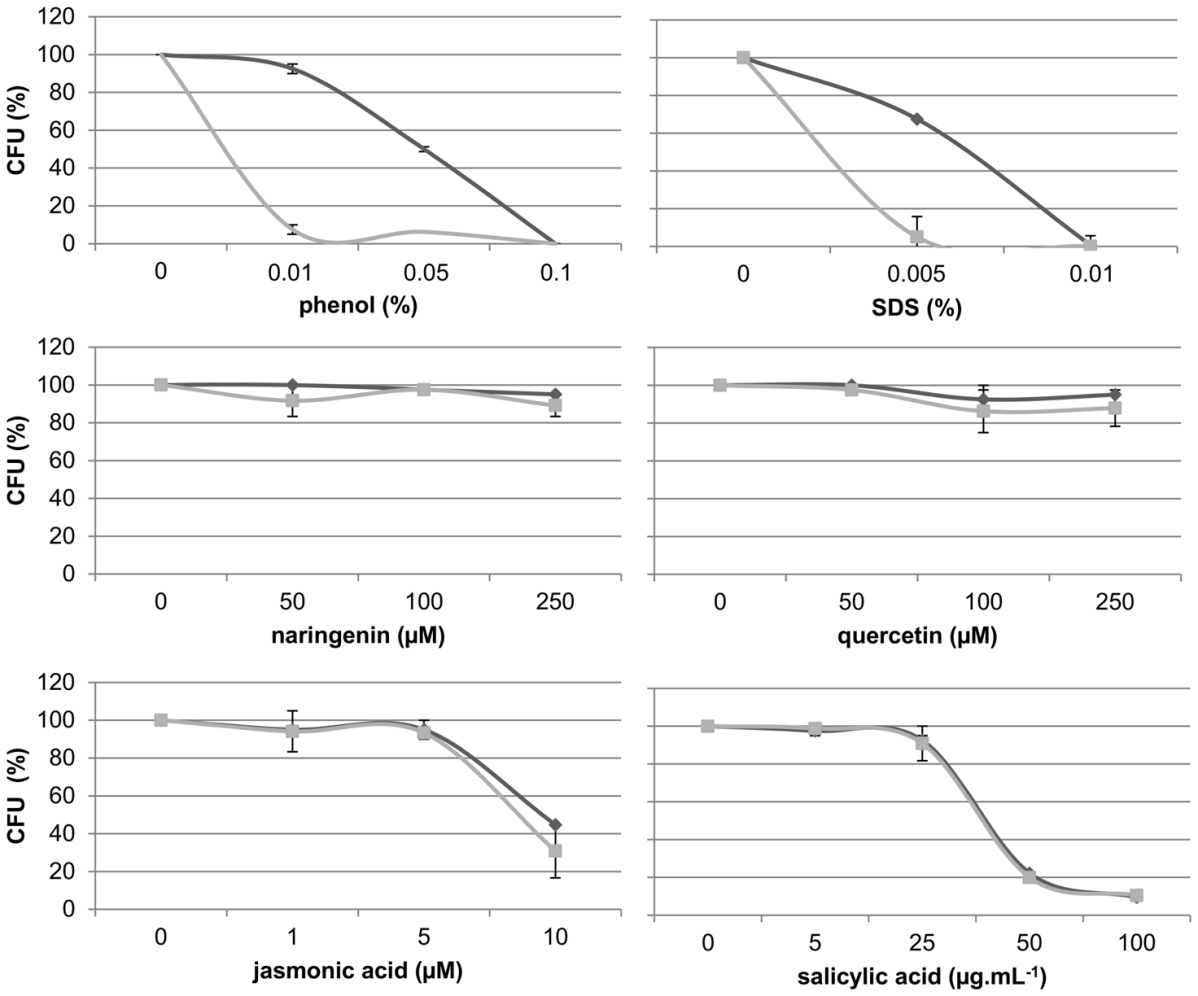
Resistance of *H. seropedicae* strains to chemical stress. *H. seropedicae* wild type (black lines) and EPSEB (gray lines) strains were plated on solid NFbHPN medium containing the compounds. Data expressed as percentage of colony forming units (CFU) in the test plates compared to the control after 24 hours of growth at 30°C.

EPS production has been associated with protection against chemical stress [3], [8], [33], [55]. We tested the resistance of the mutant and parental strain to the flavonoids naringenin and quercetin, to the plant immune metabolites jasmonic and salicylic acids, to phenol and SDS (Fig. 6). The mutant strain’s resistance to plant bactericidal compounds was not different from that of the wild type. On the other hand, the parental strain showed resistance to low concentrations of phenol and SDS, while mutation in *epsB* gene reduced the survival of the mutant strain by 95%. These results suggest that *H. seropedicae* EPS is involved in resistance to non-biochemical stress, but not in resistance to plant basal defense.

### *H. seropedicae eps* genes expression is down-regulated during maize colonization

**Figure 7:**
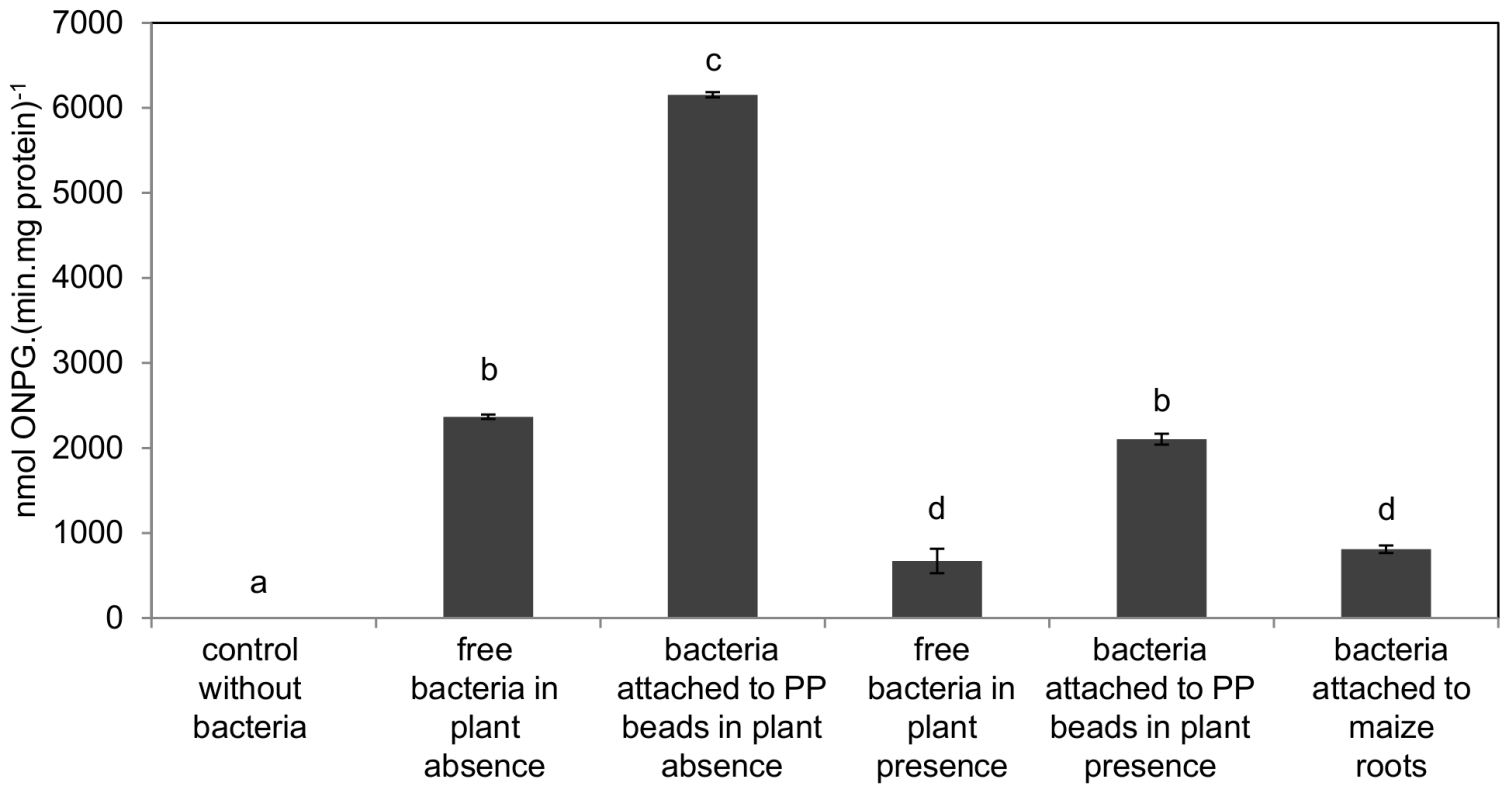
Regulation of *H. seropedicae epsG* expression during maize colonization. For maize colonization expression analyses, 10^8^ *H. seropedicae* MHS-01 (*epsG::lacZ*) cells were inoculated in the hydroponic system. After 24 hours, the cells from the hydroponic medium were collected by centrifugation. The cells attached to roots or to polypropylene spheres (PP) were removed by vortex and concentrated by centrifugation. For all the samples the β-galactosidase activity was determined, standardized by total protein concentration, and expressed as nmol ONP.(min.mg protein)^−1^± standard deviation. Different letters indicate significant differences (p<0.01, Duncan multiple range test) in *epsG* expression between the tested conditions.

Tadra-Sfeir and coworkers [38] showed by RT-PCR that the expression of *epsB* and *epsG* (code for glucosyltransferases) was repressed in the presence of the flavonoid naringenin. The reporter strain MHS01 (*epsG::lacZ*) was used to determine if the *eps* genes were regulated during maize colonization (Fig. 7). The results show that *epsG* is repressed during the first steps of interaction with maize, suggesting that EPS biosynthesis is diminished under this condition. Such repression was observed both in planktonic bacteria free in the hydroponic medium in the presence of the plant roots and in root-attached bacteria, suggesting that *H. seropedicae* EPS is not required for the attachment on root surface. On the other hand, *eps* genes were induced (2.5-fold) in the bacteria adhered to the polypropylene spheres of the hydroponic system compared to planktonic bacteria, regardless the plant presence. This result stress the involvement of *H. seropedicae* EPS in biofilm formation on inert matrices.

## Discussion

Exopolysaccharides are important factors that enable cellular aggregation and biofilm formation on solid surfaces. As shown for other plant associative bacteria [30]–[32], mutation of EPS biosynthesis genes in *H. seropedicae* SmR1 decrease EPS production and consequently biofilm formation, but surprisingly, did not alter maize colonization profile.

The importance of EPS in biofilm formation is supported by the induction of *eps* genes in the presence of inert substrates such as glass fiber and the polypropylene spheres. On the other hand, no difference was observed between the wild type and mutant strains in maize, rice or sorghum epiphytic colonization capacity. Even when lower numbers of bacteria were used to inoculate maize plants, both strains had similar root attachment patterns. Moreover, the increase and maintenance of the root epiphytic population seemed not to be dependent on EPS production. In agreement with those results, *eps* gene expression was repressed in *H. seropedicae* cells colonizing maize root surfaces. A huge impact in attachment and epiphytic colonization was observed by the lack of EPS production in *G. diazotrophicus* [32], but that seems not to be the case in *H. seropedicae* SmR1.

EPS can contribute to survival of bacteria within the plant by acting as a barrier against plant defense mechanisms, and creating a favorable microenvironment [55], [56]. EPS production seems to be important for *H. seropedicae* resistance to chemical stress caused by phenol and SDS, but not required for resistance to plant defense metabolites such as flavonoids, jasmonic and salicylic acids. Indeed, the mutant strain was able to cope with the plant chemical defense and endophytically colonize maize roots to the same extend than the wildtype. These results indicate that the product of the *eps* gene cluster is not necessary for maize root endophytic colonization by *H. seropedicae*.

The results lead us to propose a model for the early steps of *H. seropedicae* maize colonization. Upon contact with the rhizosphere environment *eps* genes are down-regulated, decreasing EPS biosynthesis. On the other hand, LPS biosynthesis is up-regulated, which allows the bacteria to bind to plant lectins on the root surface [46]. In accordance with this suggestion, scanning electron microscopy [34], [35] and the CLSM results showed that *H. seropedicae* cells form a monolayer on maize root surface, not developing to mature biofilm. It seems that *H. seropedicae* biofilm development is arrested on roots by the reduced biosynthesis of EPS. The loosely attached bacterial cell can then penetrate inner root tissues and colonize them. By avoiding permanent attachment and biofilm maturation *H. seropedicae* would remain available to seek penetration sites and nutrient sources.

In most plant-interacting bacteria studied so far, including associative, symbiotic or pathogenic, whenever the EPS is involved in biofilm formation it is also required for plant colonization or acts as a virulence factor [32], [55]–[73]. In a stark contrast, *H. seropedicae* SmR1 EPS is necessary for biofilm formation but EPS synthesis is repressed during maize root colonization.

## Supporting Information

Figure S1 ***H. seropedicae*** **SmR1** ***eps*** **gene cluster.** The proteins coded by the showed genes were analyzed in Table S1. The indicated probable promoter regions were identified with the BPROM software (SoftBerry).
(TIFF)

Figure S2 **Electrophoretic pattern of LPS isolated from** ***H. seropedicae*** **SmR1 (A) and EPSEB (B).** SDS-PAGE was performed with total LPS extracted from 10^7^ cells grown in NFbHPN medium by the SDS/proteinase K method, and visualized with silver periodate oxidation staining.
(TIFF)

Figure S3 **Rice (A) and sorghum (B) root colonization by** ***H. seropedicae*** **wild type (black bars) and** ***epsB*** **(gray bars) mutant strain.** Results are shown as average of Log_10_ (number of bacteria.g^−1^ of fresh root) ± standard deviation. d.a.i. = days after inoculation.
(TIFF)

Table S1 ***H. seropedicae*** **Eps proteins.**
(DOC)
