# Supplementary figures and images for: Exopolysaccharide Biosynthesis Enables Mature Biofilm Formation on Abiotic Surfaces by Herbaspirillum seropedicae
Source: PLoS One. 2014 Oct 13;9(10):e110392. doi: 10.1371/journal.pone.0110392 (PMC4195743; doi:10.1371/journal.pone.0110392)

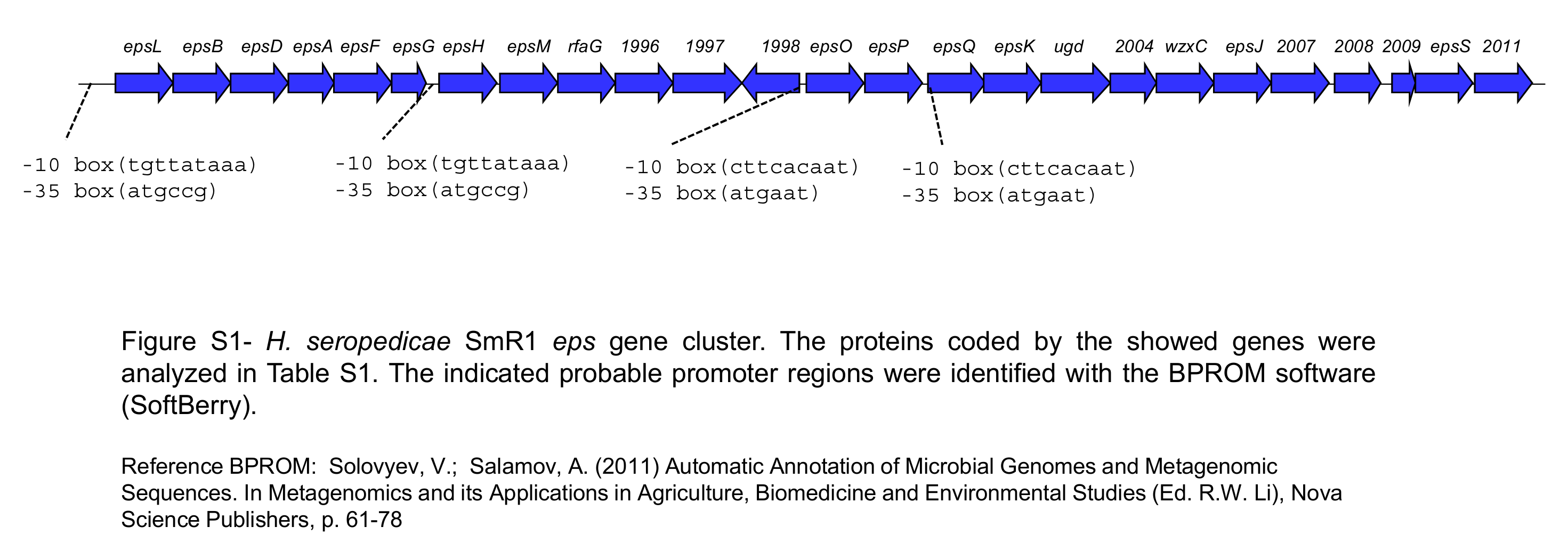

Supplement: Figure S1 — H. seropedicae SmR1 eps gene cluster. The proteins coded by the showed genes were analyzed in Table S1. The indicated probable promoter regions were identified with the BPROM software (SoftBerry). (TIFF) [file pone.0110392.s001.tiff]

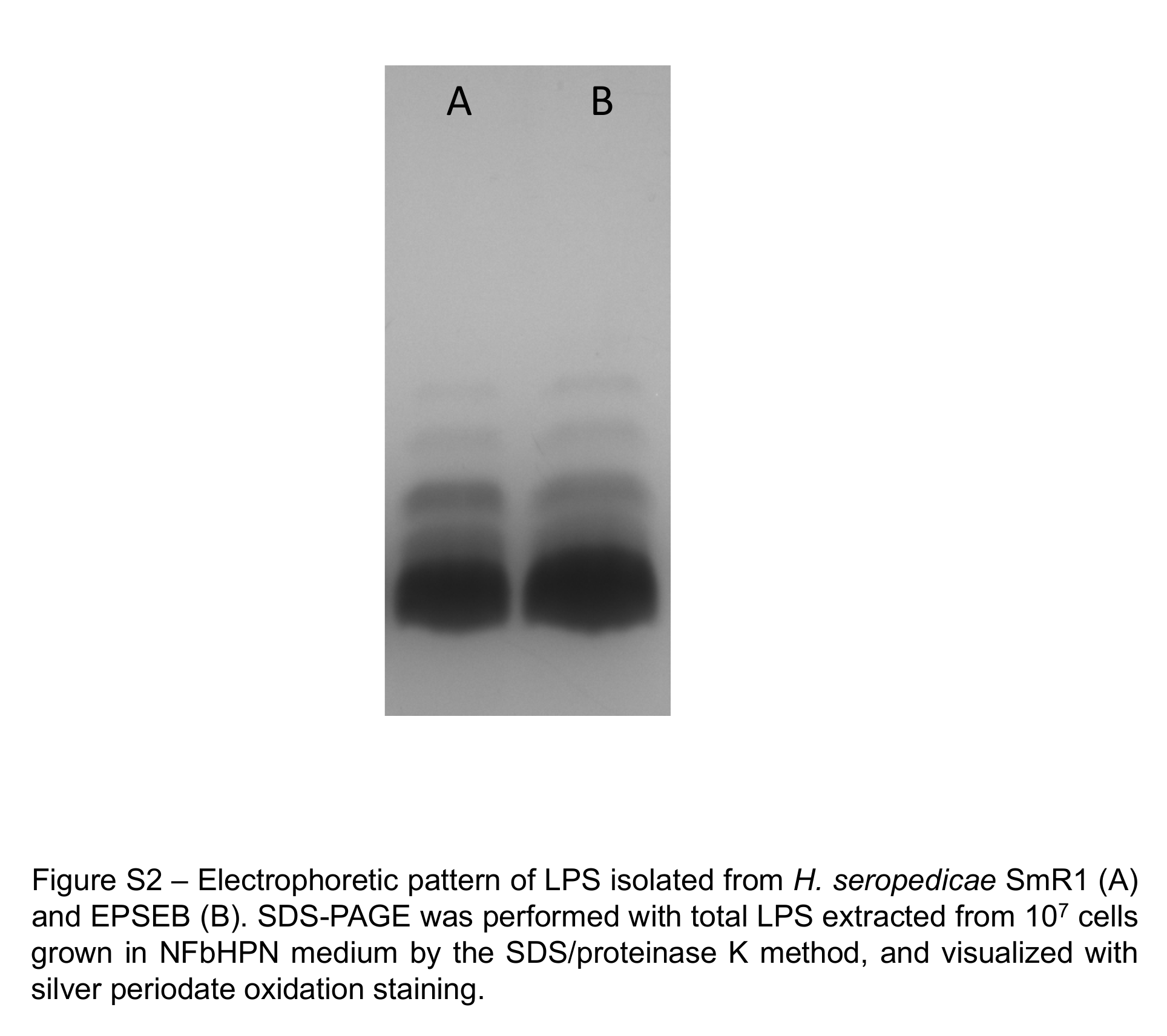

Supplement: Figure S2 — Electrophoretic pattern of LPS isolated from H. seropedicae SmR1 (A) and EPSEB (B). SDS-PAGE was performed with total LPS extracted from 107 cells grown in NFbHPN medium by the SDS/proteinase K method, and visualized with silver periodate oxidation staining. (TIFF) [file pone.0110392.s002.tiff]

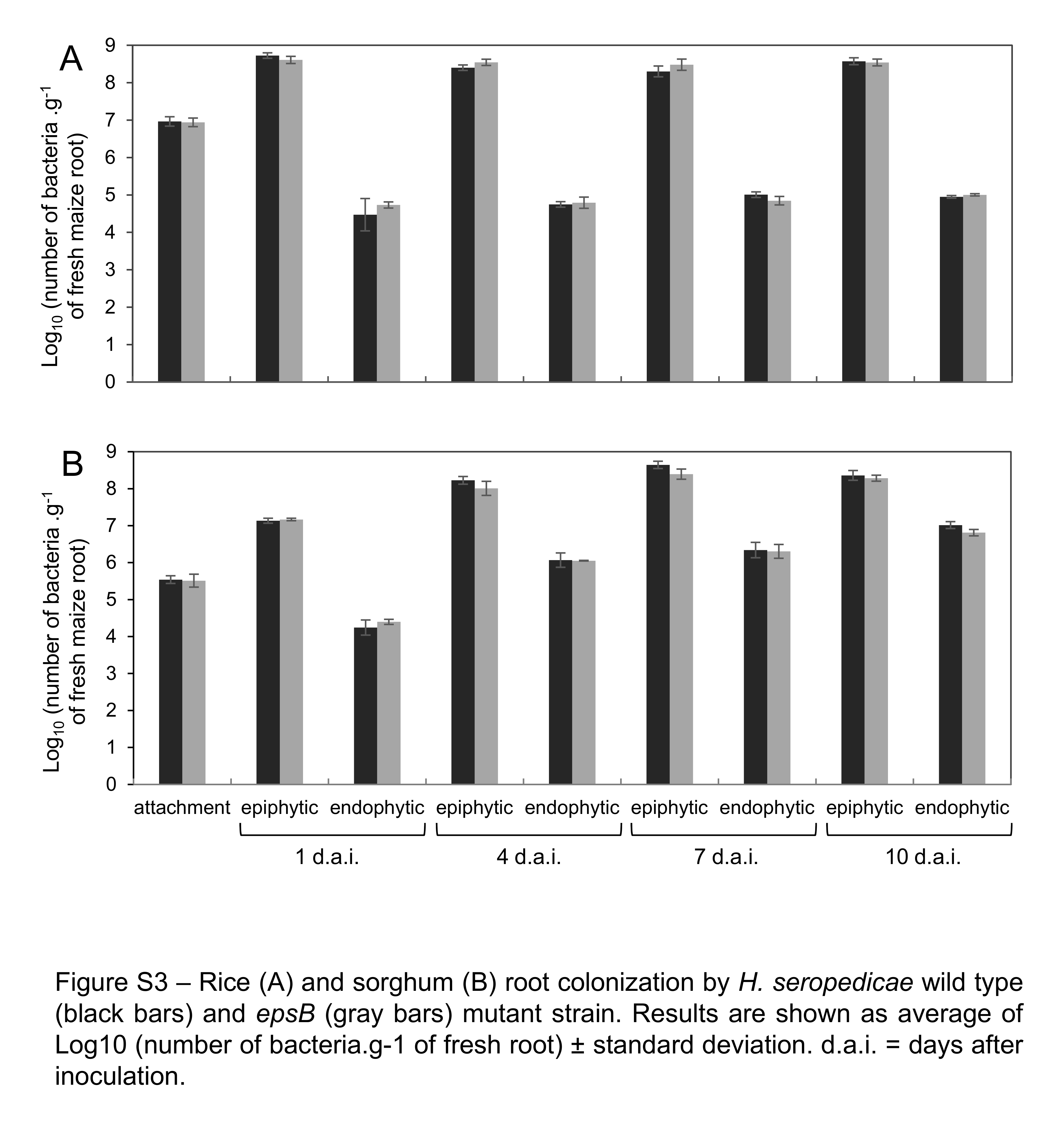

Supplement: Figure S3 — Rice (A) and sorghum (B) root colonization by H. seropedicae wild type (black bars) and epsB (gray bars) mutant strain. Results are shown as average of Log10 (number of bacteria.g−1 of fresh root) ± standard deviation. d.a.i. = days after inoculation. (TIFF) [file pone.0110392.s003.tiff]
