# Supplementary material for: Exopolysaccharide Biosynthesis Enables Mature Biofilm Formation on Abiotic Surfaces by Herbaspirillum seropedicae
Source: PLoS One. 2014 Oct 13;9(10):e110392. doi: 10.1371/journal.pone.0110392 (PMC4195743; doi:10.1371/journal.pone.0110392)
Supplement: Table S1 — H. seropedicae Eps proteins. (DOC) [file pone.0110392.s004.doc]

Table S1 – *H. seropedicae* Eps proteins

| **Proteins from *H. seropedicae*** | **Homologs** | **Identity/ Similarity (%) ^a^** | **Proposed function** | **Reference ^b^** |
| --- | --- | --- | --- | --- |
| EpsL | Hear_0712 [*Herminiimonas arsenicoxydans*]  EpsL [*Methylobacillus* sp. 12S] | 40 / 56  25 / 40 | EPS biosynthesis | |YP_001099033.1|  |BAC55133.1| |
| EpsB | EpsB [*Methylobacillus* sp. 12S] | 53 / 72 | Glucosyltransferase | |BAC41337.1| |
| EpsD | EpsD [*Methylobacillus* sp. 12S] | 31 / 58 | Peptidyl-prolyl cis-trans isomerase | |BAC55134.1| |
| EpsA | EpsE [Methylobacillus sp. 12S] | 39 / 61 | Polysaccharide export outer membrane protein | |BAC55135.1| |
| EpsF | EpsF [Methylobacillus sp. 12S] | 47 / 69 | Chain length determinant protein | |BAC55136.1| |
| EpsG | EpsG *Oxalobacteraceae bacterium* IMCC9480 | 59 / 76 | Glucosyltransferase | |EGF32585.1| |
| EpsH | EpsH *Oxalobacteraceae bacterium* IMCC9480 | 54 / 68 | Exosortase 2 | |EGF32586.1| |
| EpsM | Mma_0637 *Janthinobacterium* sp. Marseille | 50 / 69 | EPS biosynthesis | |YP_001352327.1| |
| RfaG | Glycoside hydrolase Pseudomonas putida H8234 | 67 / 77 | Glucosyltransferase | |AGN79251.1| |
| Hsero_1996 | Acyltransferase Bradyrhizobium sp. ORS 278 | 49 / 64 | Acyltransferase | |YP_001206761.1| |
| Hsero_1997 | Acyltransferase Acidovorax sp. MR-S7 | 42 / 56 | O-antigen acetylase | |GAD21245.1| |
| Hsero_1998 | Mma_0639 Janthinobacterium sp. Marseille | 58 / 70 | Conserved hypothetical | |YP_001352329.1| |
| EpsO | Hear_0724 *Herminiimonas arsenicoxydans* | 53 / 68 | Glucosyltransferase | |YP_001099045.1| |
| EpsP | Hear_0725  *Herminiimonas arsenicoxydans* | 56 / 69 | Glucosyltransferase | |CAL60919.2| |
| EpsQ | CpsB  *Herminiimonas arsenicoxydans* | 70 / 83 | GDP-mannose pirofosforilase | |CAL60922.2| |
| EpsK | Mpq_1819  *Methylovorus* sp. MP688 | 43 / 58 | Glucosyltransferase | |YP_004040208.1| |
| Ugd | Ugd  *Ralstonia solanacearum* PSI07 | 73 / 84 | UDP-glucose 6-dehydrogenase | |YP_003753104.1| |
| Hsero_2004 | *Sphingobium chlorophenolicum* L-1 | 44 / 61 | Glucosyltransferase | |ZP_07574652.1| |
| WzxC | WzxC *Oxalobacteraceae*  *bacterium* IMCC9480 | 61 / 79 | Polysaccharide flippase | |EGF32588.1| |
| EpsJ | Bphy_6724 *Burkholderia phymatum* STM815 | 43 / 61 | Glucosyltransferase | |YP_001862793.1| |
| Hsero_2007 | Acyltransferase Pseudomonas protegens Pf-5 | 34 / 50 | Acyltransferase | |YP_261319.1| |
| Hsero_2008 | No hits |  | Hypothetical |  |
| Hsero_2009 | O-acetyltransferase Herminiimonas arsenicoxydans | 68 / 82 | O-antigen acetyltransferase | |YP_001099043.1| |
| EpsS | GalE *Janthinobacterium* sp. Marseille | 71 / 85 | UDP-glucose 4-epimerase | |YP_001352334.1| |
| Hsero_2011 | Hear_0718 *Herminiimonas arsenicoxydans* | 68 / 80 | Glucosyltransferase | |YP_001099039.1| |

^a^ Protein alignment performed with NCBI PSI-Blastp (http://ncbi.nlm.gov/BLAST/).

^b^ Database access number.
